# FieldSimR: an R package for simulating plot data in multi-environment field trials

**DOI:** 10.3389/fpls.2024.1330574

**Published:** 2024-04-04

**Authors:** Christian R. Werner, Dorcus C. Gemenet, Daniel J. Tolhurst

**Affiliations:** ^1^Accelerated Breeding Initiative (ABI), Consultative Group of International Agricultural Research (CGIAR), Texcoco, Mexico; ^2^International Maize and Wheat Improvement Center (CIMMYT), Texcoco, Mexico; ^3^The Roslin Institute and Royal (Dick) School of Veterinary Studies, University of Edinburgh, Edinburgh, United Kingdom

**Keywords:** simulation, spatial variation, plot error, multi-environment field trials, linear mixed models

## Abstract

This paper presents a general framework for simulating plot data in multi-environment field trials with one or more traits. The framework is embedded within the R package FieldSimR, whose core function generates plot errors that capture global field trend, local plot variation, and extraneous variation at a user-defined ratio. FieldSimR’s capacity to simulate realistic plot data makes it a flexible and powerful tool for a wide range of improvement processes in plant breeding, such as the optimisation of experimental designs and statistical analyses of multi-environment field trials. FieldSimR provides crucial functionality that is currently missing in other software for simulating plant breeding programmes and is available on CRAN. The paper includes an example simulation of field trials that evaluate 100 maize hybrids for two traits in three environments. To demonstrate FieldSimR’s value as an optimisation tool, the simulated data set is then used to compare several popular spatial models for their ability to accurately predict the hybrids’ genetic values and reliably estimate the variance parameters of interest. FieldSimR has broader applications to simulating data in other agricultural trials, such as glasshouse experiments.

## 1 Introduction

This paper presents a general framework for simulating plot data in multi-environment field trials with one or more traits. The framework is embedded within the R package FieldSimR, whose core function generates plot errors that capture global field trend, local plot variation, and extraneous variation. FieldSimR’s capacity to simulate realistic plot data makes it well-suited to a wide range of improvement processes in plant breeding, such as the optimisation of experimental designs and statistical analyses of multi-environment field trials. It is also well-suited to a range of education purposes, such as teaching the principals of spatial modelling and multi-environment trial analysis.

Plant breeding programmes continuously evaluate, select, and release improved genotypes in order to meet the complex and dynamic requirements of different customer groups, including farmers, processors, and end-users (Covarrubias-Pazaran et al., 2022). The resources required to compare different improvement strategies in the field, however, can quickly exceed the practical possibilities of a plant breeding programme. Often, multiple factors must be evaluated simultaneously over several years or even decades to identify an optimised breeding strategy. This requires a pragmatic approach to identify profitable long-term strategies in plant breeding programmes.

Simulation is a fast and cost-efficient tool for comparing different breeding strategies over time (Gaynor et al., 2021). Interestingly, this is not a new concept. Simulations have been utilised by plant and animal breeders for almost a century, beginning with the application of the Breeder’s equation (Lush, 1937), a form of deterministic simulation to predict genetic gain based on selection intensity, selection accuracy, genetic variance, and generation interval. However, only recently, with the availability of modern computers and flexible software have breeders and researchers been granted access to more powerful stochastic simulations for optimising entire breeding programmes across multiple generations. Currently available software includes QU-GENE (Podlich and Cooper, 1998), ADAM-plant (Liu et al., 2019), and ChromaX (Younis et al., 2023), as well as the R packages Selection Tools (Frisch, 2023) and AlphaSimR (Gaynor et al., 2021). These software applications can be used, for example, to compare different crossing and selection strategies over time. They lack, however, the functionality to simulate realistic plot data in multi-environment field trials. This capacity is necessary to evaluate the impact of different experimental designs, multi-environment testing strategies, and statistical analyses on the performance of a breeding programme.

The motivation to simulate realistic plot data has stemmed from the importance of spatial variation in plant breeding field trials (see, for example, Wilkinson et al., 1983; Besag and Kempton, 1986; Cullis and Gleeson, 1991; Rodríguez-Álvarez et al., 2018; Piepho et al., 2022). Spatial variation occurs naturally in field trials laid out as a two-dimensional lattice of plots (Gogel et al., 2023), and can account for more than 50% of the total phenotypic variation. Spatial variation can be broadly categorised as either global trend, local variation, or extraneous variation (Gilmour et al., 1997). Global trend occurs on a large scale across the field, such as large scale moisture and fertility gradients (Green et al., 1985). Local variation occurs on a small scale between neighbouring plots. It may reflect small scale changes in soil composition (trend) or random error (noise), such as measurement error and within-plot variability (Besag, 1977). Conversely, extraneous variation is predominately induced during the conduct of the trial, and as a result is often aligned with the column and row dimensions. It may reflect management practices, such as serpentine harvesting and spraying, multi-plot seeders that sow multiple plots simultaneously, or inaccurate trimming resulting in unequal plot lengths (Stefanova et al., 2009). The complexity and importance of spatial variation dictate the need for a general framework to simulate realistic plot errors that capture the main components described above.

FieldSimR is an R package for simulating plot errors in multi-environment field trials that capture global and local trend, random error, and extraneous variation. It also provides compatibility with AlphaSimR to generate plot phenotypes, by combining the simulated plot errors with genetic values. This makes FieldSimR a powerful tool for a wide range of improvement processes, such as:

- Comparing spatial modelling approaches, e.g., separable autoregressive processes, tensor-product penalised splines, and nearest neighbour adjustments.
- Comparing experimental designs for single- and multi-environment studies, e.g., complete and incomplete block designs, including various *p*-rep and sparse testing designs.
- Comparing approaches for analysing multi-environment trial data, e.g., reaction norms, random regressions, and factor analytic models.

The paper is arranged as follows. The “Methods” section presents the theoretical framework for simulating plot errors, which are generated by combining spatial, random, and extraneous error components at a user-defined ratio. The “Results and discussion” section introduces an example simulation of field trials that evaluate 100 maize hybrids for two traits in three environments. To demonstrate FieldSimR’s value as an optimisation tool, the simulated data set is then used to compare several popular spatial models for their ability to accurately predict the hybrids’ genetic values and reliably estimate the variance parameters of interest.

## 2 Methods

This section presents the framework in FieldSimR for simulating plot errors in multi-environment field trials. FieldSimR generates plot errors by combining spatial, random, and extraneous error components at a user-defined ratio. The simulation framework is initially developed for a single trait and then extended for multiple traits.

### 2.1 Framework for simulating plot errors in multi-environment field trials

Assume a single-trait multi-environment trial data set comprises *p* environments with *n* plots in total, where $n=\sum_{j=1}^{p}n_{j}$ and $n_{j}$ is the number of plots in environment *j*. Also assume that each environment is laid out as a two-dimensional lattice of plots such that *n_j_* = *c_j_* × *r_j_*, where *c_j_* and *r_j_* are the number of columns and rows, respectively. The *n*-vector of plot errors is then given by ${\varepsilon =(\varepsilon_{1}^{\text{T}},\ldots ,\varepsilon_{p}^{\text{T}})}^{\text{T}}$ , where ***ε**_j_* is the *n_j_*-vector of plot errors for environment *j* (ordered as rows within columns). The vector ***ε**_j_* captures the main components of spatial variation, i.e., global and local trend, random error, and extraneous variation.

FieldSimR generates the vector of plot errors for each environment as the sum of three terms:


$$\varepsilon_{j}=s_{j}+r_{j}+e_{j},$$

where **s***_j_* is a vector of errors that capture global and local spatial trend, **r***_j_* is a vector of random errors, and **e***_j_* is a vector of errors that capture extraneous variation. The errors in **s***_j_* and **e***_j_* are hereafter referred to as the spatial and extraneous errors, respectively. All terms are simulated as mutually independent with zero means and variance components given by $\sigma_{s_{j}}^{2}$ , $\sigma_{r_{j}}^{2}$ , and $\sigma_{e_{j}}^{2}$ , respectively. The total plot error variance is then given by $\sigma_{\varepsilon_{j}}^{2}=\sigma_{s_{j}}^{2}+\sigma_{r_{j}}^{2}+\sigma_{e_{j}}^{2}$ .

#### 2.1.1 Spatial error

The errors in **s***_j_* capture both global and local trend, such as large scale fertility gradients (Green et al., 1985) and small scale changes in soil composition (Gilmour et al., 1997). FieldSimR has the capacity to generate spatial errors based on either bivariate interpolation (Akima, 1978) or an autoregressive process (Box and Jenkins, 1970). The key difference is that bivariate interpolation applies a (non-stochastic) smoothing function to the errors, while autoregressive processes assume correlated errors based on a stochastic variance matrix (Gogel et al., 2023). Both approaches have been widely and successfully adopted for the empirical analysis of field trial data, and hence their implementation within FieldSimR.

**Figure 1:**
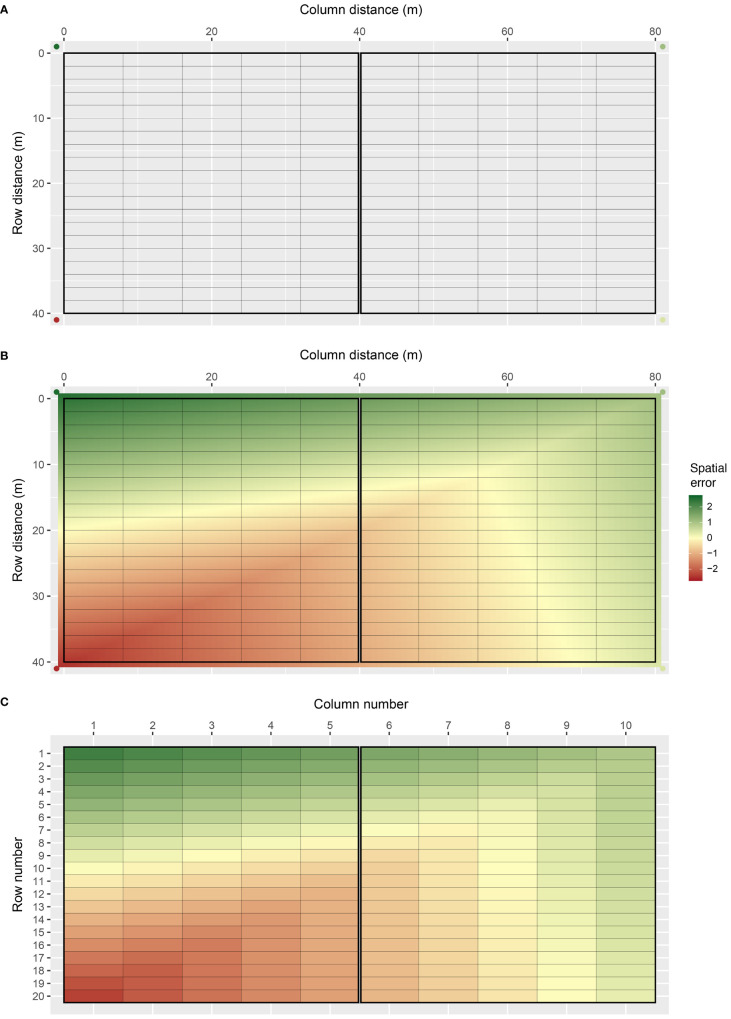
Demonstration of how FieldSimR generates spatial errors using bivariate interpolation: **(A)** the two-dimensional lattice of plots is constructed with four knot points placed outside the four corners, **(B)** the continuous array between the knot points is interpolated which produces a smooth continuous surface, and **(C)** a single error value is assigned to each plot by averaging over the continuous surface within each plot.

Bivariate interpolation is implemented through the interp() function in the R package interp (Gebhardt et al., 2023), which applies piece-wise linear interpolation across the two-dimensional lattice of plots. An example field array with spatial trend generated using bivariate interpolation is presented in **Figure 1**. The field array comprises *c_j_* = 10 columns and *r_j_* = 20 rows for *n_j_* = 200 plots in total. The field spans 80 m long in the column direction and 40 m wide in the row direction, with rectangular plots 8 m long by 2 m wide (**Figure 1A**). There are two square blocks aligned in the column direction (side-by-side), with 100 plots in each block. Four interpolation (knot) points are placed outside the four corners of the field, which prevents continuity issues that occur at the interpolation boundary. The *z*-values at these points were sampled from a standard normal distribution, with *z* = 2.56, 1.08, 0.43, and −2.56 for the example (clockwise from top left). The continuous array between the knot points is then interpolated, which produces a smooth continuous surface across the lattice of plots (**Figure 1B**). A single error value is assigned to each plot by averaging over the continuous surface within each plot (**Figure 1C**). The error values are then scaled to the defined spatial error variance for each environment, $\sigma_{s_{j}}^{2}$ . This produces the vector of spatial errors, **s***_j_*.

**Figure 2:**
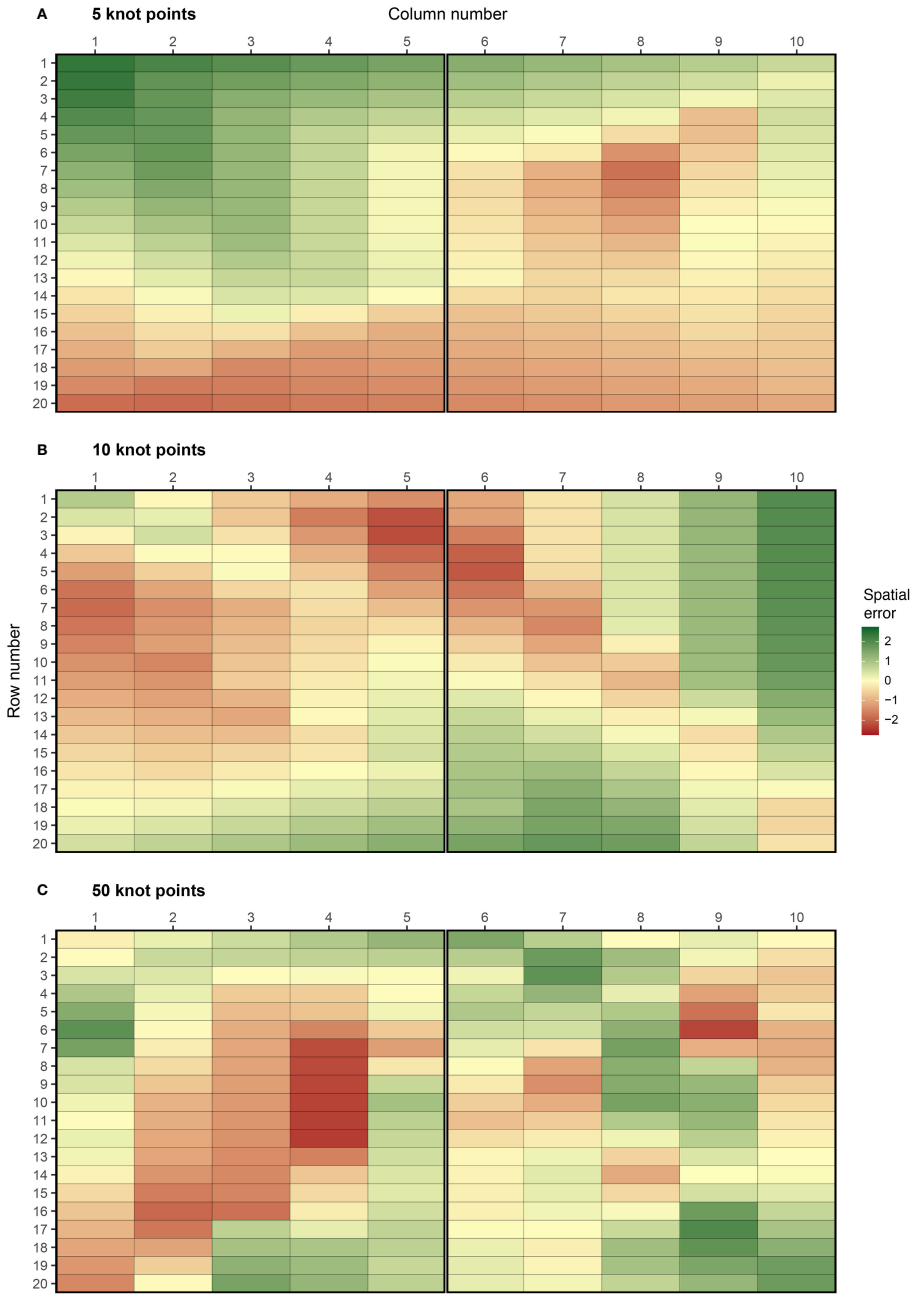
Examples of spatial errors generated using bivariate interpolation with **(A)** 5, **(B)** 10, and **(C)** 50 knot points. These options are set using complexity = 5, 10, and 50. The coordinates of the knot points are presented in **Supplementary Figure S2**. The graphics were produced with the plot_effects() function in FieldSimR.

The complexity of spatial trend can be controlled in FieldSimR by setting the number of additional knot points sampled inside the field array. By altering the complexity, users can explicitly change the ratio of global to local trend. The example in **Figure 1** has no additional knot points besides those at the four corners, so the simulated spatial error predominately captures global trend with minimal or no local trend. Three additional examples are presented in **Figure 2**, which have 5, 10, and 50 knot points, respectively. The knot points are sampled from a continuous uniform distribution defined by all points in the continuous array. This means that more than one knot point can be sampled for each plot. The position of the knot points and corresponding *z*-values are presented in **Supplementary Figure S1**, which displays the smooth continuous surface for the examples in **Figure 2**.

The examples demonstrate FieldSimR’s capacity to simulate global and local trend, as well as within-plot variability. Increasing the complexity will generate more local trend relative to global trend, up to a point where the errors capture minimal or no trend (i.e., only noise). At this point, numerous knot points may be sampled for each plot which further increases the amount of within-plot variability. By default, FieldSimR sets the number of knot points to half the maximum of the number of columns and rows. For example, the default complexity for a field trial with 10 columns and 20 rows is given by max(10, 20)/2 = 10 knot points (see, for example, **Figure 2B**). This generally provides a good ratio of global to local trend, but users are encouraged to alter the complexity as required.

Trellis plots for the three examples are presented in **Supplementary Figure S2**. These graphics also demonstrate that various ratios of global to local trend can be generated by altering the complexity. For example, the first graphic demonstrates a gradual decrease in spatial error as the row number increases, which is a classical sign of global trend in field trials. Conversely, the last graphic demonstrates more small-scale fluctuations between neighbouring columns and rows, which is a sign of local trend. Note that since bivariate interpolation is a smoothing function, rather than a stochastic process, the spatial errors are not simulated as random variables.

Alternatively, the spatial errors can be generated as random variables in FieldSimR based on a separable first order autoregressive (AR1) process. Separable AR1 processes explicitly model spatial dependence (correlation) between neighbouring plots in the column and row dimensions, rather than interpolating a smooth continuous surface across the field array. In this case, FieldSimR simulates the vector of spatial errors:


$$s_{j}∼\text{N}\left(0,\sigma_{s_{j}}^{2}S_{j}\right),$$

where $\sigma_{s_{j}}^{2}$ is the spatial error variance and **S***_j_* is the *n_j_* ×*n_j_* separable correlation matrix, which is constructed as:


$$S_{j}={\text{Σ}}_{c_{j}}\left(\rho_{c_{j}}\right)⊗{\text{Σ}}_{r_{j}}\left(\rho_{r_{j}}\right),$$

where $\rho_{c_{j}}$ is the column autocorrelation parameter with *c_j_* × *c_j_* correlation matrix ${\text{Σ}}_{c_{j}}$ and $\rho_{r_{j}}$ is the row autocorrelation parameter with *r_j_* × *r_j_* correlation matrix ${\text{Σ}}_{r_{j}}$ . Note that, in contrast to bivariate interpolation, the separable AR1 process does not require plot dimensions, since they are implicitly modelled through $\rho_{c_{j}}$ and $\rho_{r_{j}}$ (see Gilmour et al., 1997). This approach allows users to implement estimates of $\rho_{c_{j}}$ and $\rho_{r_{j}}$ previously obtained from empirical analyses of field trial data.

The ratio of global to local trend can be controlled by altering the column and row autocorrelation parameters. Decreasing the autocorrelation parameters will effectively increase the complexity of the spatial trend, in the sense that more local trend will be generated relative to global trend, up to a point where the errors capture minimal or no trend (i.e., only noise). This occurs when the autocorrelation parameters are set to zero. Three examples are presented in **Supplementary Figure S3**, which show spatial trend generated using a separable AR1 process with (a) $\rho_{c_{j}}$ = 0.7 and $\rho_{r_{j}}$ = 0.9, (b) $\rho_{c_{j}}$ = 0.5 and $\rho_{r_{j}}$ = 0.7, and (c) $\rho_{c_{j}}$ = 0.3 and $\rho_{r_{j}}$ = 0.5. The theoretical and sample variograms for these examples are presented in **Supplementary Figure S4**. The examples demonstrate the stochastic nature of the spatial errors generated based on the separable AR1 process.

The methods above for generating global and local trend will be well-suited to most applications. However, some users may desire to explicitly set the amount of global and local trend without fine-tuning the complexity or the autocorrelation parameters. In this case, users may simulate spatial trend as the sum of two components, i.e., global trend (with no/low complexity) and local trend (with moderate/high complexity or low/moderate autocorrelations). This is left to the discretion of the user.

#### 2.1.2 Random error

The errors in **r***_j_* capture local variation that is not trend, such as noise, measurement error, and intrinsic variability within the plots (Besag, 1977; Wilkinson et al., 1983). FieldSimR simulates the vector of random errors as:


$$r_{j}∼\text{N}\left(0,\sigma_{r_{j}}^{2}I_{n_{j}}\right),$$

where $\sigma_{r_{j}}^{2}$ is the random error variance and $I_{n_{j}}$ is an identity matrix of order *n_j_*.

#### 2.1.3 Extraneous error

The errors in **e***_j_* capture extraneous variation predominately induced during the conduct of the trial, such as serpentine harvesting or spraying and unequal plot dimensions (Gilmour et al., 1997; Stefanova et al., 2009). This type of variation is assumed to be aligned with the columns and rows of the trial. FieldSimR generates the vector of extraneous errors as the sum of two terms as:


$$e_{j}=Z_{c_{j}}e_{c_{j}}+Z_{r_{j}}e_{r_{j}},$$

where $e_{c_{j}}$ is a vector of column errors with *n_j_* × *c_j_* design matrix $Z_{c_{j}}$ and $e_{r_{j}}$ is a vector of row errors with *n_j_* × *r_j_* design matrix $Z_{r_{j}}$ . The design matrices are given by Zcj=Icj⊗1rj and Zrj=1cj⊗Irj .

The column and row errors are simulated as:


$$e_{c_{j}}∼\text{N}\left(0,\sigma_{e_{c_{j}}}^{2}I_{c_{j}}\right)\text{ and }e_{r_{j}}∼\text{N}\left(0,\sigma_{e_{r_{j}}}^{2}{\text{I}}_{r_{j}}\right),$$

where $\sigma_{e_{c_{j}}}^{2}$ is the column error variance and $\sigma_{e_{r_{j}}}^{2}$ is the row error variance, which are set according to whether column and/or row errors are desired. The total extraneous error variance is then given by $\sigma_{e_{j}}^{2}=\sigma_{e_{c_{j}}}^{2}+\sigma_{e_{r_{j}}}^{2}$ .

**Figure 3:**
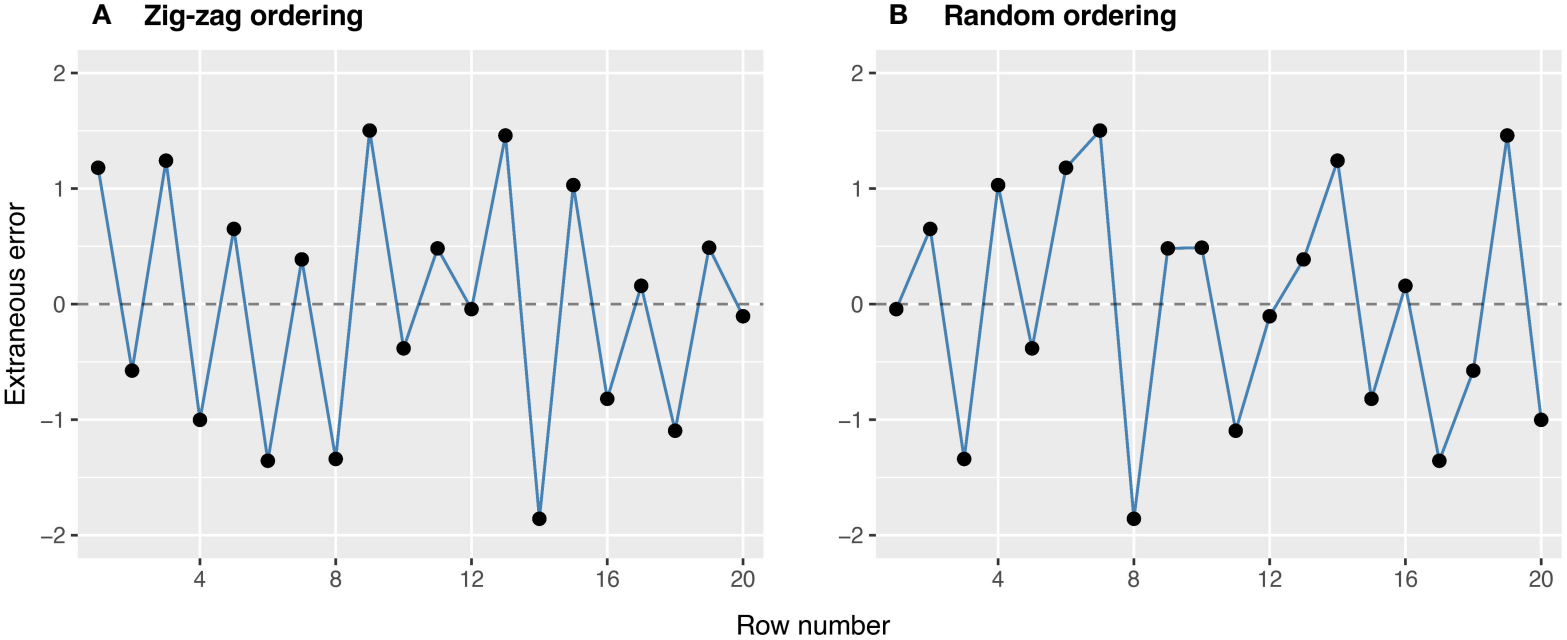
Examples of extraneous errors generated using **(A)** zig-zag and **(B)** random ordering. These options are set using ext.ord = “zig-zag” and “random”.

FieldSimR has the capacity to simulate extraneous errors based on zig-zag or random ordering between neighbouring columns and/or rows. The zig-zag ordering is achieved by alternating the positive and negative simulated values between neighbouring columns and rows. The two examples in **Figure 3** demonstrate the two types of extraneous variation. The first example demonstrates a zig-zag pattern where the errors in odd row numbers are always positive (mean of +0.37), while those in even row numbers are always negative (mean of -0.37). This type of non-stationarity is a classical sign of extraneous variation attributed to systematic management practices, such as serpentine harvesting and spraying. The second example demonstrates a more stochastic pattern in which the errors may be attributed to random processes, such as inaccurate plot trimming resulting in unequal plot dimensions. Interested users may also manipulate the above functionality to simulate interplot competition, typically observed as a negative correlation between neighbouring rows (Durban et al., 2001; Stringer et al., 2011).

#### 2.1.4 Total plot error

FieldSimR generates the total plot errors in Equation 1 by combining the spatial errors with the random and extraneous errors according to a user-defined ratio. The desired ratio is applied by setting the proportions of spatial and extraneous error variance, with the remaining proportion assigned to random error. By default, FieldSimR sets the proportion of spatial error to 0.5 and extraneous error to 0, resulting in a proportion of random error variance of 0.5.

### 2.2 Extension to multiple traits

FieldSimR has the capacity to simulate correlated plot errors for multiple traits. The correlation matrix between traits can be set for the spatial, random, and extraneous errors terms separately. By default, FieldSimR fits a separable correlation structure between traits and environments (Bančič et al., 2023), but note that different error variances can be set for different environment-within-trait combinations. It is also important to note that when bivariate interpolation is used, the correlation matrix for the spatial errors is applied to the *z*-values at the knot points, not the spatial errors themselves. This is because the spatial errors generated using bivariate interpolation do not have an assumed stochastic variance matrix, and the *z*-values are treated as the random variables.

## 3 Results and discussion

FieldSimR is an R package for simulating plot errors that comprise global and local trend, random error, and extraneous variation. This functionality makes FieldSimR a powerful tool for a wide range of improvement processes, such as the comparison of different spatial modelling approaches. This section demonstrates the simulation and analysis of field trials that evaluate 100 maize hybrids for two traits in three environments. In the first part, FieldSimR is used to simulate plot errors, genetic values, and phenotypes for the 100 maize hybrids in the three field trials. In the second part, eight spatial models are compared for their ability to accurately predict the true genetic values of the maize hybrids and to reliably estimate the true variance parameters of interest.

### 3.1 Simulation example

Consider a scenario in which 100 maize hybrid genotypes are evaluated for grain yield (t/ha) and plant height (cm) in field trials across three environments. The simulation of plot phenotypes with FieldSimR involves three steps:

1. Simulation of plot errors.
2. Simulation of genetic values.
3. Generation of phenotypes by combining the plot errors with the genetic values.

#### 3.1.1 Simulation of plot errors

Plot errors for grain yield and plant height were simulated with FieldSimR’s core function field_trial_error(), assuming independence between traits and between environments. Environments 1 and 2 comprised two blocks each, while Environment 3 comprised three blocks. The blocks were aligned in the column direction (side-by-side) and comprised 5 columns and 20 rows for 100 plots in each block. The plots were 8 m long in the column direction by 2 m wide in the row direction.

To obtain plot-level heritabilities of h^2 ^= 0.3 for grain yield and h^2 ^= 0.5 for plant height in all three environments, the total error variances for the two traits were defined relative to their genetic variances, as demonstrated in **Supplementary Script S10**. The simulated plot errors comprised spatial, random, and extraneous error terms. The spatial errors were simulated using bivariate interpolation with complexity set to 10 and proportion of spatial error variance set to 0.4 in all three environments. The extraneous errors were simulated using zig-zag ordering across neighbouring rows. The proportion of extraneous error variance was set to 0.2 in all three environments. This resulted in a proportion of random error variance of 1 − (0.4 + 0.2) = 0.4.


error_df <- field_trial_error(ntraits = 2,
             nenvs = 3,
             nblocks = c(2,2,3),
             ncols = c(10,10,15),
             nrows = 20,
             block.dir = "col",
             varR = c(0.20, 0.28, 0.14,
             15.1, 8.5, 11.7),
             spatial.model = "Bivariate",
             complexity = 10,
             plot.length = 8,
             plot.width = 2,
             prop.spatial = 0.4,
             ext.ord = "zig-zag"
             ext.dir = "row",
             prop.ext = 0.2)

**Figure 4:**
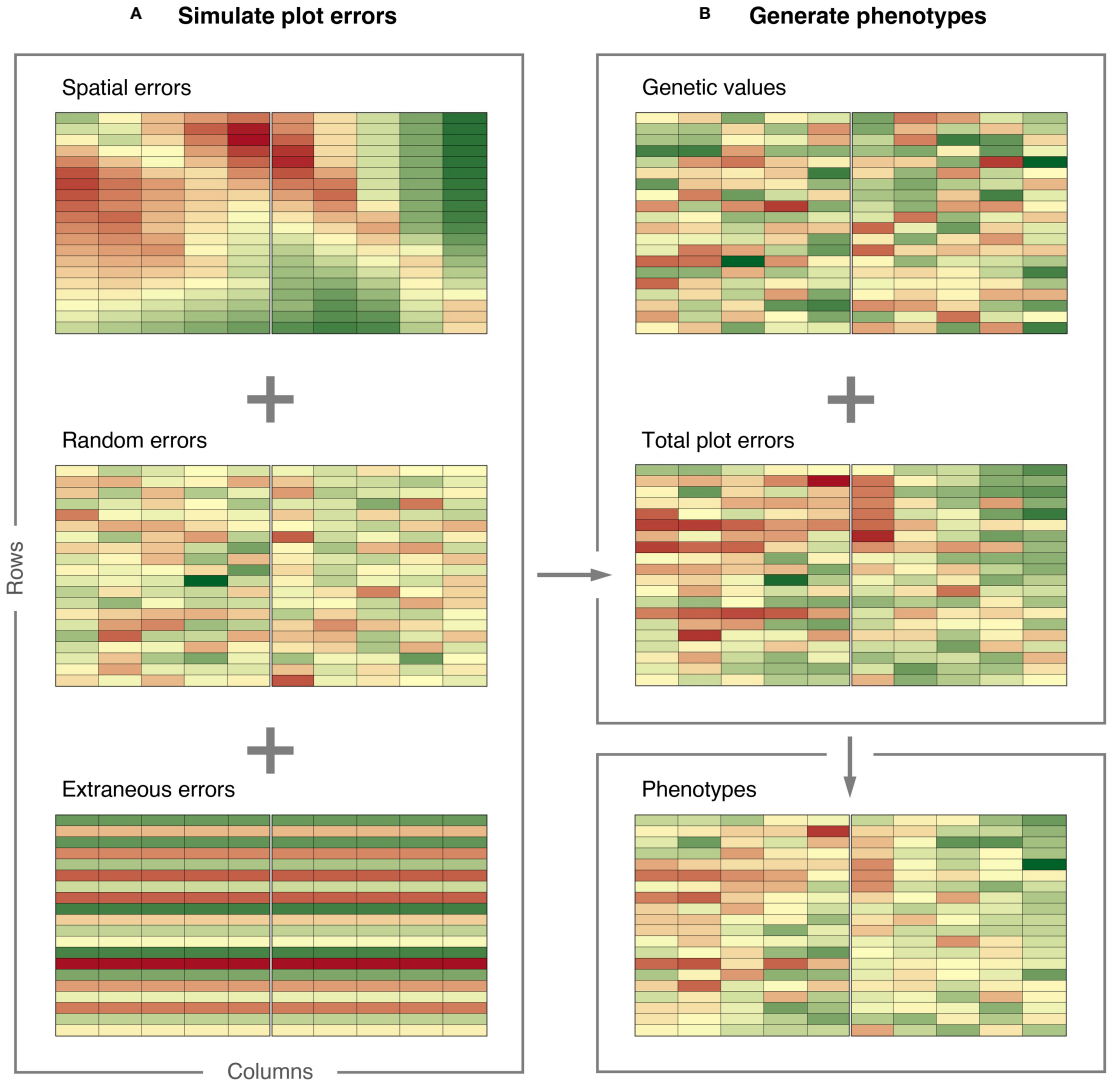
Demonstration of how FieldSimR generates phenotypes: **(A)** plot errors are simulated by combining the spatial errors with the random and extraneous errors at a user-defined ratio, and **(B)** phenotypes are generated by combining the total plot errors with the true genetic values simulated with AlphaSimR. The graphics were produced with the plot_effects() function in FieldSimR.

The simulated plot errors can also be accessed through the example data frame error_df_bivar, which will be used below to generate phenotypes. The spatial errors, extraneous errors, random errors, and total plot errors are presented in **Figure 4** for gain yield in Environment 1.

#### 3.1.2 Simulation of genetic values

Genetic values for grain yield and plant height in the three environments were simulated based on an unstructured model for genotype-by-environment (GxE) interaction. The simulation was done in AlphaSimR (Gaynor et al., 2021), using FieldSimR’s wrapper functions unstr_asr_input() and unstr_asr_output(), as demonstrated in **Supplementary Script S10**. The simulated genetic values can be accessed through the example data frame gv_df_unstr. The genetic values for grain yield in Environment 1 are presented in **Figure 4**.

In addition to the unstructured model, FieldSimR provides wrapper functions for simulating genetic values based on compound symmetry and multiplicative models for GxE interaction. Alternatively, users can provide their own set of genetic values, e.g., through simulation or previously obtained from empirical analyses.

#### 3.1.3 Generation of phenotypes

Phenotypes for grain yield and plant height were generated by combining the simulated plot errors with the genetic values stored in FieldSimR’s example data frame gv_df_unstr. The maize hybrids were randomly allocated to plots according to a randomised complete block design (RCBD).


pheno_df <- make_phenotypes(gv.df = gv_df_unstr,
              error.df = error_df_bivar,
              randomise = TRUE)

The phenotypes are presented together with the plot errors and genetic values in **Figure 4** for grain yield in Environment 1. The graphics in **Figure 4** were produced with FieldSimR’s plot function plot_effects().


plot_effects(df = pheno_df[pheno_df$env == 1,],
        effect = "y.Trait1")

FieldSimR currently provides functionality to generate an RCBD, but note that other (incomplete) designs are being implemented. Users will also have the ability to apply experimental designs generated externally, e.g., with R packages such as agricolae (de Mendiburu, 2023), odw (Butler, 2021), and DiGGer (Coombes, 2020).

### 3.2 Comparison of spatial models

The comparison of spatial models is demonstrated for the simulated grain yield data in Environment 1 (**Figure 4**). A sequential approach was adopted for model fitting following Gilmour et al. (1997), with global trend and extraneous variation diagnosed using the sample variogram and accounted for using fixed and random model terms. This resulted in eight spatial models, including a baseline model, three models with a separable first order autoregressive (AR1) process, two models with a tensor-product penalised spline (TPS), and two models implementing nearest neighbour (NN) adjustments (**Table 1**). All models were fitted with ASReml-R (Butler et al., 2018), as demonstrated in **Supplementary Script S11**.

**Table 1:** Linear mixed models fitted to the simulated grain yield data in **Figure 4**, Part 1: Summary of non-genetic model terms.

		Fixed			Random				
Model	Terms	Col	Row	Col:Row	Col	Row	AR1	TPS	ID
Baseline	ID								✓
1	AR1						✓		
2	AR1 + ID						✓		✓
3	AR1 + ID + Frow + Rrow		✓			✓	✓		✓
4	TPS + ID	✓	✓	✓				✓	✓
5	TPS + ID + Rcol + Rrow	✓	✓	✓	✓	✓		✓	✓
6	NN + ID								✓
7	NN + ID								✓

All models included a fixed overall mean, and random genotype and block effects. The separable AR1 process included one variance and two autocorrelation parameters. The TPS included three fixed and five random spline terms, each with their own variance parameter. The moving grids used in the NN adjustments are presented in **Supplementary Figure S9**. AR1, first order autoregressive; TPS, tensor-product penalised spline; NN, nearest neighbour; ID, independent error.

The spatial models were evaluated in three ways (**Table 2**):

1. The model fit was assessed using the residual maximum likelihood ratio test (REMLRT) and the Akaike information criterion (AIC).
2. The prediction accuracy was calculated as Pearson’s correlation coefficient (*r*) between the true genetic values from the simulation and the predicted genetic values from the analysis.
3. The bias was calculated as the difference between the true and estimated genetic variance parameters.

**Table 2:** Linear mixed models fitted to the simulated grain yield data in **Figure 4**, Part 2: Model fit criteria, prediction accuracy, and bias.

Model	Fixed	Pars	-2 loglik	REMLRT	AIC	Accuracy	Bias
Baseline	1	3	−70.4		−66.7	0.65	−0.026
1	1	5	−99.1	*p* < 0.0001	−94.4	0.72	−0.014
2	1	6	−113.7	*p* < 0.0001	−103.7	0.74	−0.021
**3**	**2**	**7**	−133.4		−126.6	**0.76**	−0.012
4	4	8	−110.8		−93.8	0.69	−0.050
**5**	**4**	**10**	−135.7	***p* < 0.0001**	−116.7	**0.72**	−0.021
**6**	**1**	**3**	−103.8		−102.0	**0.71**	** −0.003 **
7	1	3	−94.2		−94.7	0.70	−0.023

Presented for each model are the number of fixed terms and autocorrelation/variance parameters, residual deviance, REMLRT, AIC, prediction accuracy, and bias. The selected AR1, TPS, and NN models are distinguished with *bold font*. The REMLRT is applied sequentially and cannot compare models with different fixed effects. Models 6 and 7 cannot be compared with REMLRT and AIC because the phenotypes have been adjusted. AR1, first order autoregressive; TPS, tensor-product penalised spline; NN, nearest neighbour; ID, independent error.

Note that the expected prediction accuracy for the data set is 0.68, based on the simulation parameters. Also note that the REMLRT is based on the non-zero variance approach of Stram and Lee (1994) and the AIC is based on the full log-likelihood approach of Verbyla (2019), which can compare models with different fixed effects. Typical experimental design and data checks were performed prior to model fitting (**Supplementary Figure S5**).

#### 3.2.1 Baseline model

The analyses commenced by fitting a baseline linear mixed model, which included random genotype and block effects, and an independent (ID) error term (**Table 1**). This model reflects a classical complete block analysis that assumes independent genotypes, blocks, and residuals. The prediction accuracy of the baseline model was lower than the expected accuracy (*r* = 0.65 compared to 0.68; **Table 2**). The estimated genetic variance was ${\hat{\sigma }}_{g}^{2}=0.061$ , which was lower than the true value of 0.087 (bias = −0.026; **Tables 2**, **3**).

**Table 3:** Linear mixed models fitted to the simulated grain yield data in **Figure 4**, Part 3: REML estimates of autocorrelation and variance parameters.

	Genotype	Block	Col	Row	AR1			TPS					ID
Model	${\hat{\sigma }}_{g}^{2}$	${\hat{\sigma }}_{b}^{2}$	${\hat{\sigma }}_{e_{c}}^{2}$	${\hat{\sigma }}_{e_{r}}^{2}$	${\hat{\sigma }}_{s}^{2}$	${\hat{\rho }}_{c}$	${\hat{\rho }}_{r}$	${\hat{\sigma }}_{s_{1}}^{2}$	${\hat{\sigma }}_{s_{2}}^{2}$	${\hat{\sigma }}_{s_{3}}^{2}$	${\hat{\sigma }}_{s_{4}}^{2}$	${\hat{\sigma }}_{s_{5}}^{2}$	${\hat{\sigma }}_{r}^{2}$
Baseline	0.061	0.02											0.19
1	0.073	0.00			0.20	0.51	0.23						
2	0.066	0.00			0.40	0.95	0.87						0.09
**3**	**0.075**	**0.00**		**0.01**	**0.10**	**0.75**	**0.89**						**0.08**
4	0.037	0.05						0.03	0.03	0.69	0.00	0.07	0.16
**5**	**0.066**	**0.04**	**0.00**	**0.05**				**0.04**	**0.02**	**0.70**	**0.00**	**0.08**	**0.09**
**6**	**0.084**	**0.00**											**0.14**
7	0.064	0.00											0.17

The selected AR1, TPS, and NN models are distinguished with *bold font*. The separable AR1 process included one variance and two autocorrelation parameters. The TPS included five random spline terms, each with their own variance parameter. The true genetic variance was $\sigma_{g}^{2}=0.087$ , while the true error variances were $\sigma_{s}^{2}=0.08$ , $\sigma_{e_{r}}^{2}=0.04$ , and $\sigma_{r}^{2}=0.08$ . AR1, first order autoregressive; TPS, tensor-product penalised spline; NN, nearest neighbour; ID, independent error.

#### 3.2.2 Separable first order autoregressive processes

Models 1, 2, and 3 included random genotype and block effects, and a separable AR1 process. The separable AR1 process represents a stochastic process which assumes correlated residuals in the column and row dimensions (Martin, 1990; Cullis and Gleeson, 1991). It comprised one variance and two autocorrelation parameters (**Table 1**).

Model 1 provided a significantly better fit than the baseline model in terms of REMLRT (*p <* 0.0001) and AIC (−94.4 compared to −66.7), and produced a higher prediction accuracy (*r* = 0.72 compared to 0.65; **Table 2**). The estimated genetic variance was ${\hat{\sigma }}_{g}^{2}=0.073$ , which provided a better estimate than the baseline model (bias = –0.014; **Tables 2**, **3**). The estimated column and row autocorrelations were ${\hat{\rho }}_{c}=\;0.51$ and ${\hat{\rho }}_{r}=\;0.23$ .

**Figure 5:**
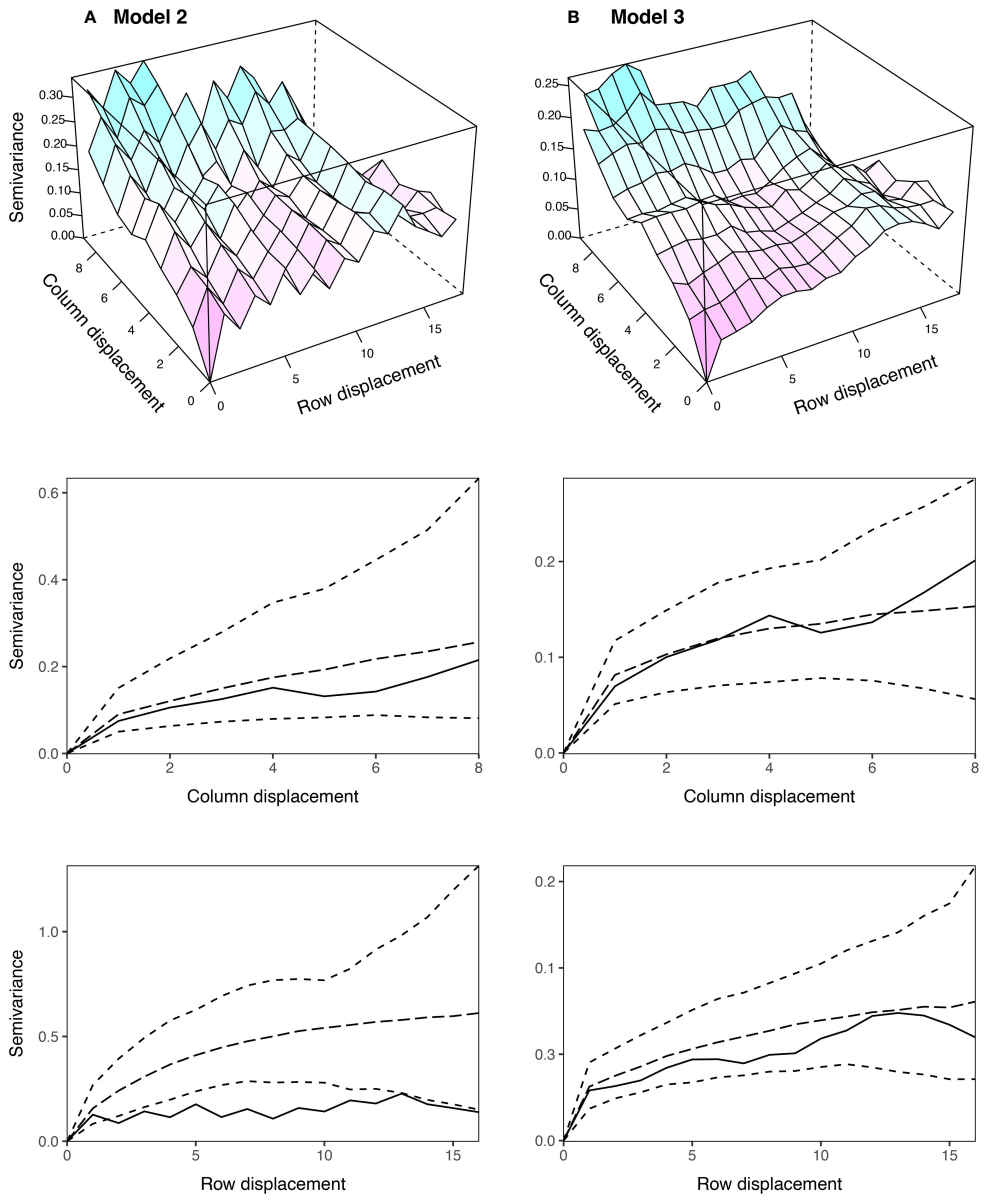
Sample variograms for the AR1 spatial models fitted to the simulated grain yield data in **Figure 4**: **(A)** Model 2: AR1 + ID and **(B)** Model 3: AR1 + ID + Frow + Rrow. The column and row faces of each variogram were constructed following Stefanova et al. (2009), and are supplemented with approximate 95% coverage intervals. Only semivariances based on more than 30 pairs are shown. AR1, first order autoregressive process, ID, independent error, Frow, fixed row, Rrow, random row.

Model 2 was an extension of Model 1 that included an additional ID error term (Besag, 1977; Wilkinson et al., 1983). Model 2 provided a significantly better fit than Model 1 and produced a higher prediction accuracy (**Table 2**). The estimated genetic variance was ${\hat{\sigma }}_{g}^{2}=0.066$ , which was slightly lower than Model 1 (**Table 3**). The estimated column and row autocorrelations were ${\hat{\rho }}_{c}=\;0.95$ and ${\hat{\rho }}_{r}=\;0.87$ , which were substantially higher than for Model 1. This indicated that the AR1 process captured (highly correlated) spatial trend, while the ID term captured the remaining random error. The sample variogram in **Figure 5A** shows a zig-zag pattern between neighbouring rows, with consistently higher semivariances for odd displacements compared to even displacements (also see **Supplementary Figure S6**). The row face of the variogram shows that the semivariances do not fall within the coverage intervals (see Stefanova et al., 2009). This is a classical sign of extraneous variation attributed to systematic practices aligned with the rows, which matches the extraneous error simulated in this data set.

Model 3 was an extension of Model 2 that included additional fixed and random row terms. The fixed term was coded as a factor with 1 for odd row numbers and 2 for even row numbers, while the random term was coded as a factor with levels equivalent to row number (Stefanova et al., 2009). The significance of the fixed term was assessed using a Wald F-test with denominator degrees of freedom (*p <* 0.001; Kenward and Roger, 1997). Model 3 provided a better fit than Model 2 in terms of AIC and produced a higher prediction accuracy (**Table 2**). The estimated genetic variance was ${\hat{\sigma }}_{g}^{2}=\;0.075$ , which was the second best estimate of all models (**Table 3**). The estimated column autocorrelation was much lower than for Model 2 ( ${\hat{\rho }}_{c}=\;0.75$ compared to 0.95). The sample variogram in **Figure 5B** no longer shows a zig-zag pattern between neighbouring rows (also see the row face of the variogram). Instead, a discontinuity is shown at 0 displacement, reflecting the random error variance, followed by a gradual incline in the column direction. This type of non-stationarity is a sign of global trend in the column direction, which matches the spatial error simulated in this data set. However, the column face of the variogram shows that the semivariances fall within the coverage intervals. The observed non-stationarity is, therefore, an artefact of the correlated AR1 process, rather than global trend requiring further remediation. Model 3 was selected as the final AR1 spatial model based on the model fit criteria.

#### 3.2.3 Tensor-product penalised splines

Models 4 and 5 included random genotype and block effects, a TPS, and an ID error term. The TPS represents a smoothing function which is applied to the two-dimensional continuous array (Rodríguez-Álvarez et al., 2018; Piepho et al., 2022). Both models were fitted with the SpATS package (Rodríguez-Álvarez et al., 2018) and also with ASReml-R using the TPSbits helper functions (Welham, 2019). A cubic B-spline basis was used with 6 knots in the column direction and 12 knots in the row direction (Velazco et al., 2017). The TPS included three fixed and five random spline terms, each with their own variance parameter (**Table 1**).

Model 4 provided a better fit than the baseline model in terms of AIC (−93.8 compared to −66.7), and produced a higher prediction accuracy (*r* = 0.69 compared to 0.65; **Table 2**). However, Model 4 provided a poorer fit and lower prediction accuracy than any of the AR1 spatial models. The estimated genetic variance was ${\hat{\sigma }}_{g}^{2}=\;0.037$ , which provided the worst estimate of all models (bias = −0.050; **Tables 2**, **3**). Like for Model 2, the sample variogram in **Supplementary Figure S7** shows a zig-zag pattern between neighbouring rows, suggesting the presence of extraneous variation.

Model 5 was an extension of Model 4 that included additional random column and row terms. This model is equivalent to the SpATS approach of Rodríguez-Álvarez et al. (2018). Model 5 provided a significantly better fit than Model 4 and a higher prediction accuracy (**Table 2**). However, it still provided a poorer fit and lower prediction accuracy than the final AR1 spatial model, despite having five additional model parameters (**Table 1**). The sample variogram in **Supplementary Figure S8** no longer shows a zig-zag pattern, suggesting that the prescribed extraneous variation was sufficiently remediated. Model 5 was selected as the final TPS spatial model based on the model fit criteria.

#### 3.2.4 Nearest neighbour adjustments

Models 6 and 7 implemented NN adjustments to the grain yield phenotypes (Papadakis, 1937; Bartlett, 1978). The NN adjustments were obtained by averaging over neighbouring plots with the mvngGrAd package (Technow, 2015). The moving grids for Models 6 and 7 are presented in **Supplementary Figure S9**. Both models were fitted with ASReml-R, with model terms equivalent to the baseline model (**Table 1**).

Models 6 and 7 produced higher prediction accuracies than the baseline model (*r* = 0.71 and 0.70 compared to 0.65; **Table 2**). However, both models produced lower prediction accuracies than the final AR1 and TPS spatial models (*r* = 0.71 and 0.70 compared to 0.76 and 0.72, respectively). The estimated genetic variance for Model 7 was $\sigma_{g}^{2}=\;0.084$ , which was the best estimate of all models (bias = −0.003; **Table 3**). Model 7 was selected as the final NN adjusted model based on the ratio of genetic to total phenotypic variance.

## 4 Concluding remarks

FieldSimR’s capacity to simulate realistic plot errors that capture global and local trend, random error, and extraneous variation makes it a flexible and powerful tool for various improvement processes in plant breeding. It’s general framework for simulating spatial variation exploits two widely adopted approaches for analysing real-world field trial data: bivariate interpolation and autoregressive processes. In contrast to real-world data, however, FieldSimR enables the efficient and comprehensive assessment of experimental designs and statistical analyses on a large scale, across an extensive array of scenarios. It also provides a platform for obtaining unbiased comparisons of statistical approaches for their ability to accurately predict the genetic values and to reliably estimate the variance parameters of interest, as the true values are defined by the user and, therefore, are known.

FieldSimR is available on CRAN, and has been extensively deployed as part of the Excellence in Breeding (EiB) initiative to provide guidance on the improvement of field trial design and analysis strategies across numerous CGIAR breeding programmes.

## Data availability statement

The R scripts generated for this study are available in the **Supplementary Material**. FieldSimR is available to download from CRAN https://cran.rproject.org/web/packages/FieldSimR/index.html and the GitHub repository https://github.com/crWerner/fieldsimr, which also contains vignettes for simulating plot errors, genetic values, and phenotypes.

## Author contributions

CW: Conceptualization, Formal analysis, Methodology, Software, Writing – original draft, Writing – review & editing. DG: Writing – review & editing. DT: Conceptualization, Formal analysis, Methodology, Software, Writing – original draft, Writing – review & editing.
