# Supplementary material for: FieldSimR: an R package for simulating plot data in multi-environment field trials
Source: Front Plant Sci. 2024 Apr 4;15:1330574. doi: 10.3389/fpls.2024.1330574 (PMC11024423; doi:10.3389/fpls.2024.1330574)
Supplement: Supplementary file 1 [file DataSheet_1.docx]

**Supplementary Figures**

FieldSimR: An R package for simulating plot data in multi-environment field trials

Spatial
error

Supplementary Figure S1: Examples of spatial errors generated using bivariate interpolation with (a) 5,
(b) 10, and (c) 50 knot points. The coordinates of the knot points are denoted with open circles.

Supplementary Figure S2: Trellis plots of the spatial errors in Figure 2 of the manuscript generated using bivariate interpolation with (a) 5, (b) 10, and (c) 50 knot points. The coordinates of the knot points are provided in Supplementary Figure S1.

**(b)** $\boldsymbol{\rho}_{\boldsymbol{c}_{\boldsymbol{j}}}\boldsymbol{=0.5}$ **and** $\boldsymbol{\rho}_{\boldsymbol{r}_{\boldsymbol{j}}}\boldsymbol{=0.7}$

**(c)** $\boldsymbol{\rho}_{\boldsymbol{c}_{\boldsymbol{j}}}\boldsymbol{=0.3}$ **and** $\boldsymbol{\rho}_{\boldsymbol{r}_{\boldsymbol{j}}}\boldsymbol{=0.5}$

**(a)** $\boldsymbol{\rho}_{\boldsymbol{c}_{\boldsymbol{j}}}\boldsymbol{=0.7}$ **and** $\boldsymbol{\rho}_{\boldsymbol{r}_{\boldsymbol{j}}}\boldsymbol{=0.9}$

Spatial
error

Supplementary Figure S3: Examples of spatial errors generated using a separable AR1 process with column and row autocorrelations of (a) $\rho_{c_{j}}= 0.7$ and $\rho_{r_{j}}=0.9$, (b) $\rho_{c_{j}}= 0.5$ and $\rho_{r_{j}}=0.7$, and (c) $\rho_{c_{j}}= 0.3$ and $\rho_{r_{j}}=0.5$. The graphics were produced with FieldSimR’s function plot_effects().

**(a)** $\boldsymbol{\rho}_{\boldsymbol{c}_{\boldsymbol{j}}}\boldsymbol{= 0.7}$ **and** $\boldsymbol{\rho}_{\boldsymbol{r}_{\boldsymbol{j}}}\boldsymbol{=0.9}$

Semivariance

Spatial
error

**(b)** $\boldsymbol{\rho}_{\boldsymbol{c}_{\boldsymbol{j}}}\boldsymbol{= 0.5}$ **and** $\boldsymbol{\rho}_{\boldsymbol{r}_{\boldsymbol{j}}}\boldsymbol{=0.7}$

Semivariance

Spatial

error

**(c)** $\boldsymbol{\rho}_{\boldsymbol{c}_{\boldsymbol{j}}}\boldsymbol{= 0.3}$ **and** $\boldsymbol{\rho}_{\boldsymbol{r}_{\boldsymbol{j}}}\boldsymbol{=0.5}$

Semivariance

Spatial

error

Supplementary Figure S4: Theoretical and sample variograms for the spatial errors in Supplementary Figure S3 generated using a separable AR1 process with column and row autocorrelations of (a) $\rho_{c_{j}}= 0.7$ and $\rho_{r_{j}}=0.9$, (b) $\rho_{c_{j}}= 0.5$ and $\rho_{r_{j}}=0.7$, and (c) $\rho_{c_{j}}= 0.3$ and $\rho_{r_{j}}=0.5$. The graphics were produced with FieldSimR’s functions theoretical_variogram() and sample_variogram().

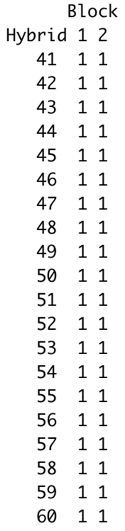

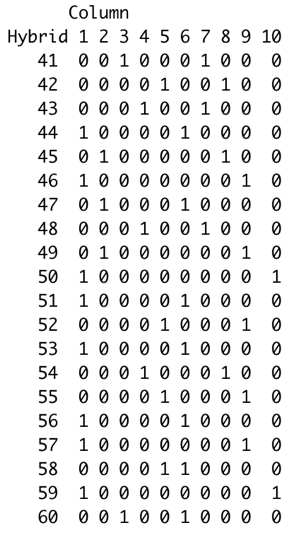

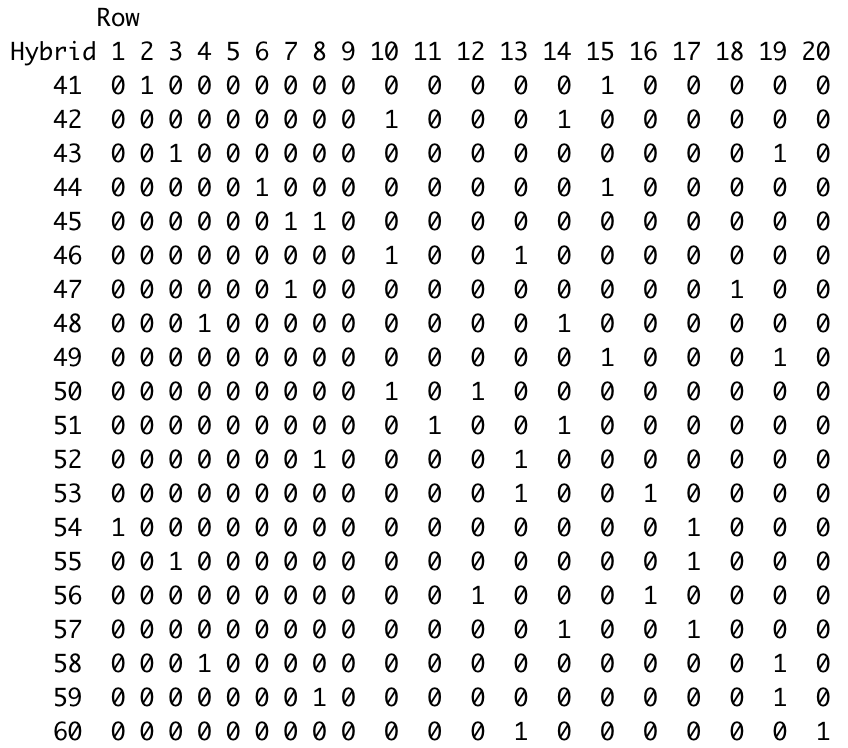

Supplementary Figure S5: Diagnostic plots for inspecting the experimental design and phenotypic data. Experimental design plots include (a) field trial layout with blocks shaded (replicate number labelled) and (b) frequency tables of hybrids in each block, column, and row (first 20 hybrids displayed for brevity). Other diagnostics include connectivity between blocks, columns, and rows (not shown). Phenotypic data plots include (c) histogram of phenotypes with theoretical distribution shown and (d) scatterplot of phenotypes grouped by hybrid (replicate number labelled).

Residual yield

Supplementary Figure S6: Diagnostic plots obtained from the AR1 spatial models. The plots include (a) trellis plot of residuals from Model 2: AR1 + ID, (b) plot of the predicted row effects from Model 3: AR1 + ID + Frow + Rrow, and (c) QQ plot of residuals from Model 3, indexed as column:row and shaded by their Studentised conditional residual (Cook et al., 1982, Thompson, 1985). AR1 - separable first order autoregressive process; ID - independent error term; Frow – fixed row term; Rrow – random row term.

Supplementary Figure S7: Diagnostic plots obtained from Model 4: TPS + ID using the (a) plot() and (b) variogram.SpATS() functions in the SpATS package (Rodríguez-Álvarez et al., 2018). TPS – tensor-product penalised spline; ID - independent error term.

Supplementary Figure S8: Diagnostic plots obtained from Model 5: TPS + ID + Rcol + Rrow using the (a) plot() and (b) variogram.SpATS() functions in the SpATS package (Rodríguez-Álvarez et al., 2018). This model is equivalent to the SpATS approach of Rodríguez-Álvarez et al. (2018). TPS – tensor-product penalised spline; ID - independent error term; Rcol – random column term; Rrow – random row term.

Supplementary Figure S9: Plots of the moving grids used in the nearest neighbour adjustments with (a) 0 and (b) 1 layers. The graphics were obtained with the sketchGrid() function in the mvngGrAd package (Technow, 2015).
